# Clinical utility of perioperative staging laparoscopy for advanced gastric cancer

**DOI:** 10.1186/1477-7819-12-350

**Published:** 2014-11-18

**Authors:** Sumiya Ishigami, Yoshikazu Uenosono, Takaaki Arigami, Shigehiro Yanagita, Hiroshi Okumura, Yasuto Uchikado, Yoshiaki Kita, Hiroshi Kurahara, Yuko Kijima, Akihiro Nakajo, Kosei Maemura, Shoji Natsugoe

**Affiliations:** Department of Digestive Surgery, and Breast and Thyroid Surgery, Kagoshima University School of Medicine, 8-35-1 Sakuragaoka, Kagoshima, 890-8520 Japan

**Keywords:** Peritoneal metastasis, Gastrectomy, Staging laparoscopy

## Abstract

**Background:**

Perioperative staging laparoscopy is a useful tool for the detection of occult peritoneal metastases in gastrointestinal cancers. This retrospective study aimed to determine the clinical value of staging laparoscopy for advanced or recurrent gastric cancer.

**Methods:**

A total of 178 patients with advanced or recurred gastric cancer who underwent perioperative staging laparoscopy were enrolled. In the absence of peritoneal deposits (P1) and positive peritoneal cytology (CY1), gastrectomy with lymph node dissection was indicated with curative intent. If P1 or CY1 was detected intraoperatively, patients received intensive chemotherapy and laparoscopic surgical intervention.

**Results:**

Curative gastrectomy was performed in 104 patients after confirmation of P0 and CY0 status. P1 or CY1 was detected for the first time in 23 (15%) patients. A total of 13 patients were converted from gastrectomy to intensive chemotherapy after detection of P1 or CY1. Additional laparoscopic interventions included insertion of intraperitoneal reservoir port in 54 patients, insertion of a metallic stent in five, ileostomy for colon stricture in six, jejunostomy in 19, and gastrojejunostomy in 16. Of eight patients treated with intensive chemotherapy who underwent R0 gastrectomy after second-look laparoscopy, five are currently free from recurrence of gastric cancer for 25.5 months.

**Conclusions:**

Occult peritoneal dissemination was detected in about 14% in patients with tumors deeper than T2. Moreover, additional laparoscopic interventions can be utilized for P1 or CY1 patients. The excellent surgical outcomes of R0 gastrectomy after chemotherapy and second-look laparoscopy indicate that confirmation of P0 and CY0 status by staging laparoscopy is of value to determine treatment strategy in patients with advanced gastric cancer.

**Electronic supplementary material:**

The online version of this article (doi:10.1186/1477-7819-12-350) contains supplementary material, which is available to authorized users.

## Background

Gastric cancer is one of the most common gastrointestinal cancers in Asian countries, especially in Japan [1]. Peritoneal dissemination (P+) is the most common type of recurrence in advanced gastric cancer, and gastric cancer with serosal exposure is often associated with P + or positive cytology (CY1) [2]. Once peritoneal metastases are detected, patient outcomes are poor despite complete (R0) resection [2–4]. The indication for cytoreductive gastrectomy in gastric cancer patients without clinical symptoms such as bleeding, strictures or perforation has been equivocal [5]. The use of chemotherapy for advanced gastric cancer has been stimulated by the development of new anticancer agents, which may provide a means of improving the clinical outcomes of gastric cancer patients with distant metastases [6–8]. We previously reported that salvage surgery in stage IV gastric cancer patients after intensive chemotherapy was associated with good clinical outcomes [9]. Therefore, detection of P1 or CY1 is an indication for intensive chemotherapy but not R0 surgery.

Although overt dissemination or ascitic fluid can be routinely detected by preoperative abdominal CT or ultrasonography combined with other modalities, tiny lesions often go undetected. The intraabdominal spread of advanced gastrointestinal cancer is often underestimated by conventional laparotomy, leading to a high rate of unnecessary laparotomies or gastrectomies [10]. Accurate tumor staging is essential for the selection of the appropriate treatment strategy for gastric cancer. In comparison to conventional extracorporeal imaging technique, staging laparoscopy is an effective and less invasive tool for the detection of unsuspected peritoneal metastasis. With the improvements in laparoscopic instruments and techniques, gastrectomy with lymph node dissection has become a popular procedure among gastrointestinal surgeons [11]. These advances have improved the accuracy of staging laparoscopy and have enabled the detection of intra-abdominal deposits by intraoperative pathological examination. Since June 2002, perioperative staging laparoscopy has been used at our institution to determine whether R0 gastrectomy or less invasive surgical intervention is indicated in patients with advanced gastric cancer. The present study is a retrospective analysis of the clinical outcomes of this series and a discussion of the clinical implications of perioperative staging laparoscopy.

## Methods

### Patients

A total of 178 gastric cancer patients who were preoperatively diagnosed with tumors deeper than T2 at Kagoshima University Hospital (Japan) were consecutively enrolled. Of the 178 gastric cancer patients, six underwent staging laparoscopy after the detection of postoperative peritoneal metastases, 71 were preoperatively suspected as having distant metastases, and the remaining 101 planned to undergo R0 gastrectomy. Type 4 tumors were diagnosed in 55 patients (Additional file 1: Table S1). Clinicopathological features were assessed using the Japanese Classification of Gastric Carcinoma (JCGC, 13th edition) [12]. This research was carried out in compliance with the Helsinki Declaration.

### Staging laparoscopy indications and procedure

**Figure 1:**
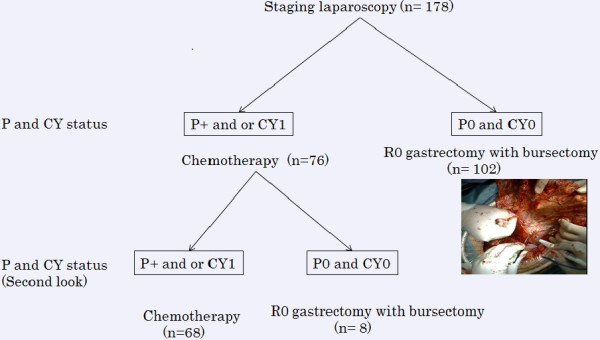
**Distribution of gastric cancer patients diagnosed by staging laparoscopy.** After the staging laparoscopy, 178 patients were divided into chemotherapy (n =76) and R0 gastrectomy (n =102) groups according to peritoneal dissemination and cytology status. Of 76 patients who received chemotherapy, eight underwent second-look laparoscopy and R0 gastrectomy.

The indications for staging laparoscopy included suspected serosal invasion of the carcinoma, the possibility of peritoneal metastases, positive cytology or the presence of ascetic fluid. Staging laparoscopy was performed under general anesthesia immediately before surgery. An incision was made at the umbilicus and a 12-mm trocar was directly introduced into the abdomen to be used as the scope port. The second and third trocars were 5 mm in diameter and were inserted at the right and left lower abdominal regions and used as the working ports. The abdominal cavity was explored systematically. First, peritoneal lavage was performed with 300 cc of saline for cytological examination of the peritoneal fluid. When the cancer cells were found in the peritoneal washing, the patient was classified as positive cytology (CY1). The condition of the peritoneum was examined. The bilateral subphrenic spaces, lesser omentum and surface of the stomach, distant peritoneum including the rectouterine pouch, and the surface of the entire bowel were first examined. Then, the omental bursa was opened and the surfaces of the pancreas and the stomach were examined. If suspicious peritoneal nodules were detected, the lesion was excised and processed into frozen sections for intraoperative histological diagnosis. When the pathological examination of the peritoneal nodule revealed adenocarcinoma, the patient was diagnosed with positivity of peritoneal metastasis (P1). In patients showing P1 and/or CY1, treatment by gastrectomy was changed to intensive chemotherapy (Figure 1). Patients with P1 and/or CY1 were mainly treated with a combination of S-1 and paclitaxel as previously reported [13].

### Treatment strategies

After confirming P0 and CY0 by staging laparoscopy, laparoscopic R0 gastrectomy plus bursectomy was performed [14]. If P1 or CY1 was identified by histological examination, gastrectomy with curative intent was discontinued and intensive chemotherapy was indicated. Furthermore, these patients received laparoscopic interventions to abate the clinical symptoms of gastric cancer. When second-look laparoscopy after intensive chemotherapy revealed neither P1 nor CY1, R0 gastrectomy was indicated (Figure 1).

### Statistical analyses

The statistical analysis of clinical features was performed using the χ2-test. Survival curves were constructed using the Kaplan-Meier method. A *P* value of <0.05 was considered significant.

## Results

### Rate of peritoneal metastases and positive cytology

**Figure 2:**
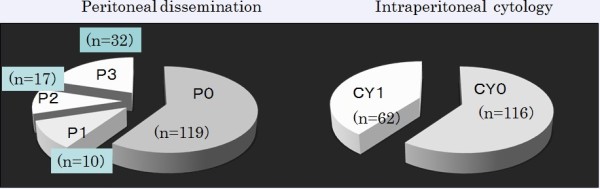
**Detection of peritoneal dissemination and positive cytology by staging laparoscopy for the first time.** Peritoneal dissemination and positivity of cytology was detected in 15% (n =23) for the first time during staging laparoscopy. P1, CY1 and P1CY1 were detected in 8, 6 and 9 patients respectively.

**Figure 3:**
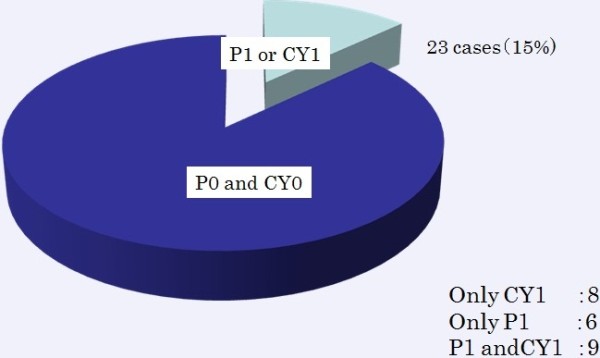
**Detection of peritoneal dissemination and positive cytology by staging laparoscopy for the first time.**

P1 and CY1 were detected in 59 (34%) and 62 (35%) patients, respectively, according to the staging laparoscopy results. P1 and/or CY1 were detected for the first time in 23 patients by laparoscopy (Figure 2). A total of 13 patients were diagnosed preoperatively, and 6, 21 and 13 patients were stage II (n =3), IIIA (n =5) and IIIB (n =5), respectively (Additional file 2: Table S2). The detection rate of P1 and/or CY1 in stage III patients was significantly higher than that in stage IB patients (*P* <0.05). P1 or CY1 was detected for the first time in 23 (15%) patients (Figure 3). Thirteen patients were converted from gastrectomy to intensive chemotherapy after detection of P1 or CY1.

### Laparoscopic interventions for P1 and/or CY1 gastric cancer patients

After the staging laparoscopy, various types of surgery were performed in different patients to reduce the clinical symptoms using a laparoscopic approach. An intraperitoneal reservoir port was inserted in 54 patients, a metallic stent was inserted in five patients, ileostomy was required in six patients for stricture of the colon, and jejunostomy and gastrojejunostomy for alimental bypass were performed in 19 and 16 patients, respectively (Additional file 3: Table S3). The cytological examination was repeated from the intraperitoneal port during intensive chemotherapy.

### R0 gastrectomy after second-look laparoscopy following intensive chemotherapy

A total of eight patients underwent R0 gastrectomy after P0 and CY0 were confirmed by second-look laparoscopy. During the follow-up period of 12 to 68 months, 3 of these patients showed peritoneal recurrence despite confirmation of P0 and CY0 status. At present, four out of five living patients are free from recurrence of carcinoma. All of the patients with recurrence of the disease showed Grade 1 histological effect of the primary tumor after chemotherapy (Additional file 4: Table S4).

## Discussion

Gastrectomy for gastric cancer is of no value in patients with P1 and/or CY1 except as a palliative surgery to reduce symptoms such as bleeding and stricture [15]. On the other hand, staging laparoscopy can be beneficial because of its high rate of detection of peritoneal metastases or positive cytology.

Song *et al*. showed that the overall accuracy of the P factor was 91.7% in T3 and T4 gastric cancer [16]. Tsuchida *et al*. also showed that the detection rate of P1 or CY1 by staging laparoscopy was higher than 30% in T4 gastric cancer [17], which is in agreement with our results. Moreover, we showed a high positivity rate of P1 or CY1 in stage III gastric cancer. Concerning cost-effectiveness [18], staging laparoscopy is indicated in patients with advanced (stage III or higher) gastric cancer.

A variety of laparoscopic interventions can be performed during staging laparoscopy and these laparoscopic interventions can especially be utilized for P1 or CY1 patients with significant clinical symptoms. Complete laparoscopic gastrojejunostomy and feeding tube insertion for gastric outlet obstruction has been reported [19–21]. In our series, 16 patients showed good recovery with food intake after gastrojejunostomy, and their quality of life was found to be improved during outpatient visits.

An intraperitoneal infuser port was inserted in 59 patients for the treatment of ascitic fluid or for the administration of anticancer agents. Furthermore, this route was repeatedly used for cytological evaluation during the course of chemotherapy. In fact, after confirmation of negative cytology using this port, R0 gastrectomy was performed after second-look laparoscopy.

In patients receiving intensive chemotherapy for P1 or CY1 gastric cancer, if lesions cannot be detected by CT or US, second-look laparoscopy should be used to estimate accurate staging. The timing of the second-look laparoscopy was generally selected based on the detection of negative cytology through the intraabdominal port. Yano *et al*. showed that a second staging laparoscopy accurately assessed the response to neoadjuvant chemotherapy, thus helping make decisions regarding R0 gastrectomy [22]. Moreover, Ajani *et al*. indicated that clinical staging by laparoscopy and endoscopic ultrasonography improved R0 resection rates after chemotherapy in patients with potentially resectable gastric carcinoma [23].

Although the surgical outcome of these patients was fairly good, three (38%) showed postoperative peritoneal recurrence. Pathological examination showed a poor histological effect of chemotherapy in these three patients. As we previously reported [9], the histological grading reflected the postoperative course of salvage surgery in the current study. When performing R0 surgery in patients with peritoneal metastasis, P0 and CY0 status should be confirmed by laparoscopy and the histological grade of the chemotherapy should be considered.

## Conclusions

In conclusion, we showed the clinical usefulness of staging laparoscopy for advanced (stage III or higher) gastric cancer patients not only to avoid negative laparotomy, but also to facilitate laparoscopic interventions. Second-look laparoscopy after intensive chemotherapy is a useful tool for confirming the indication of R0 gastrectomy.

## Electronic supplementary material

Additional file 1: Table S1: Patients information and its characteristics. (PNG 19 KB)

Additional file 2: Table S2: Changes of clinical stage after SL. (PNG 23 KB)

Additional file 3: Table S3: Surgical interventions during staging laparoscopy. (PNG 19 KB)

Additional file 4: Table S4: Case receiving R0 gastrectomy after confirmation of P0 and CY0 following intensive chemotherapy. (PNG 40 KB)

## Abbreviations

CT: Computed Tomography
CY0: negative cytology
CY1: positive cytology
P0: negative peritoneal metastases
P1: positive peritoneal metastases
R0: complete resection
US: ultrasonography.
