# Supplementary figures and images for: Clinical utility of perioperative staging laparoscopy for advanced gastric cancer
Source: World J Surg Oncol. 2014 Nov 18;12:350. doi: 10.1186/1477-7819-12-350 (PMC4247723; doi:10.1186/1477-7819-12-350)

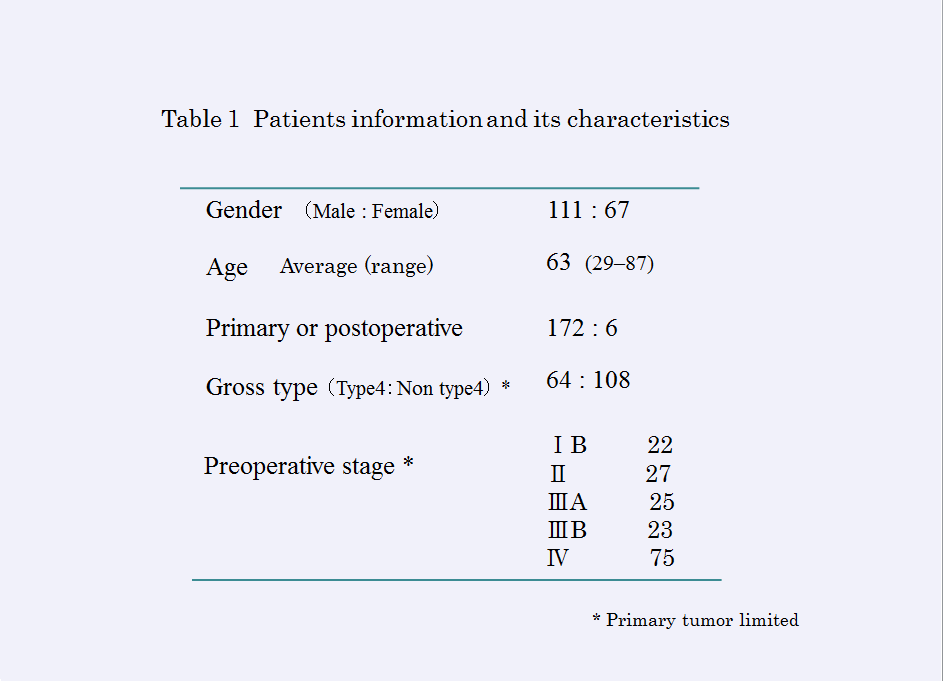

Supplement: Supplementary file 1 — Additional file 1: Table S1: Patients information and its characteristics. (PNG 19 KB) [file 12957_2013_1812_MOESM1_ESM.png]

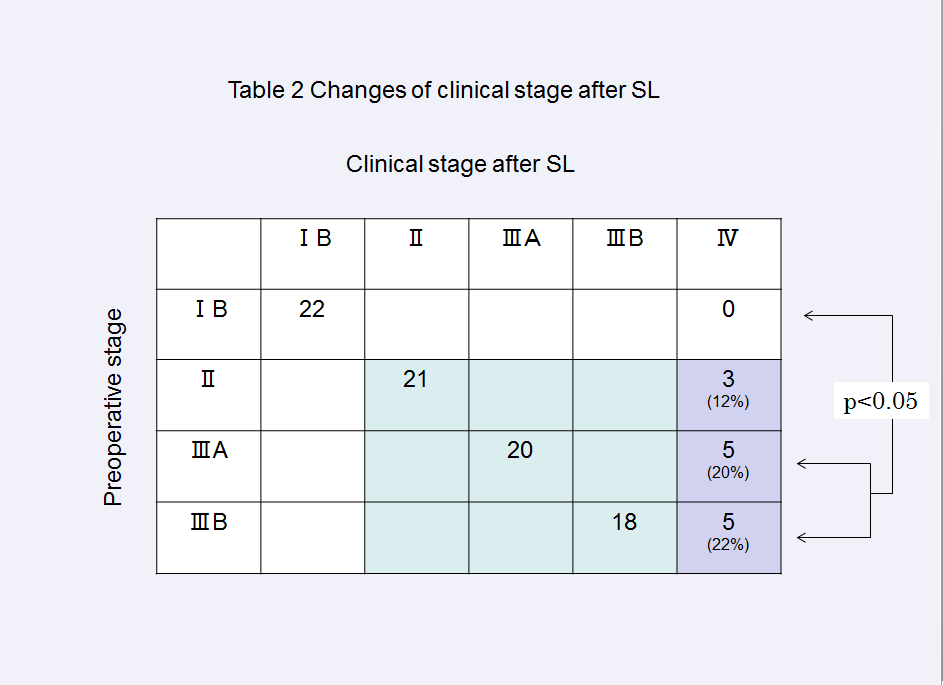

Supplement: Supplementary file 2 — Additional file 2: Table S2: Changes of clinical stage after SL. (PNG 23 KB) [file 12957_2013_1812_MOESM2_ESM.png]

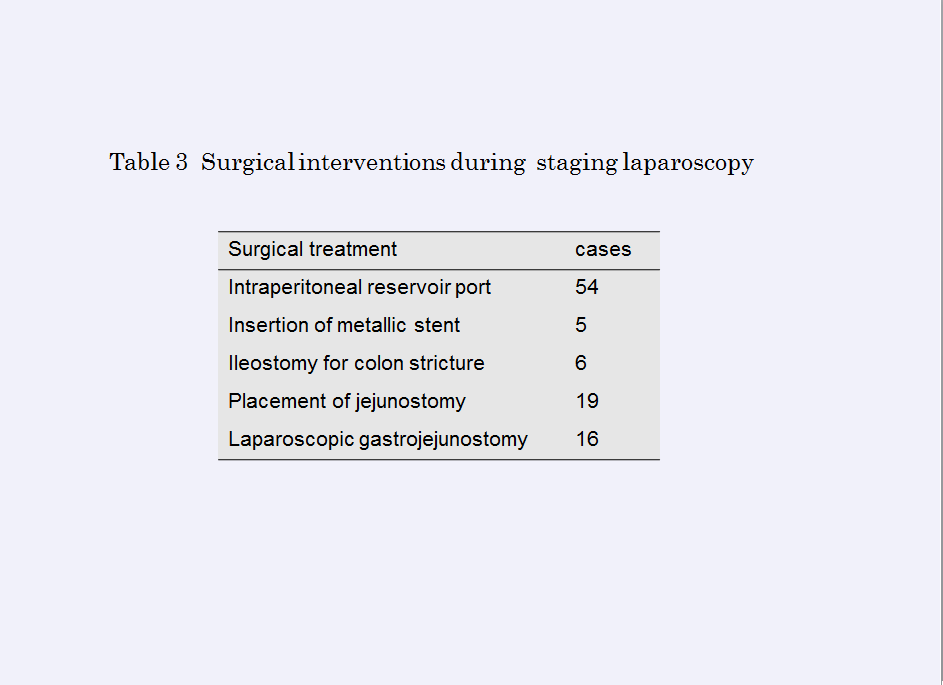

Supplement: Supplementary file 3 — Additional file 3: Table S3: Surgical interventions during staging laparoscopy. (PNG 19 KB) [file 12957_2013_1812_MOESM3_ESM.png]

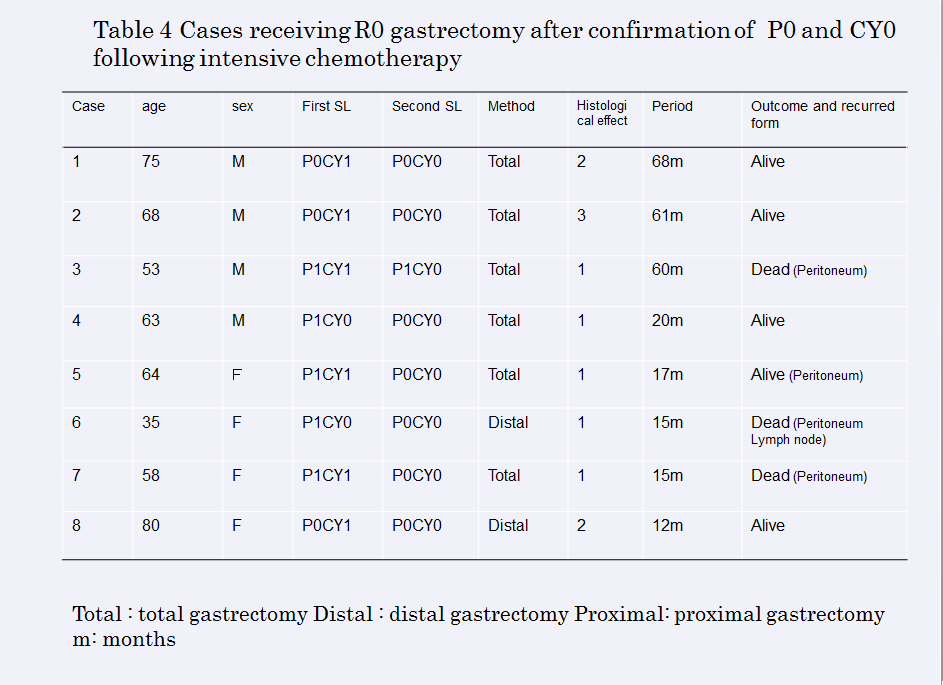

Supplement: Supplementary file 4 — Additional file 4: Table S4: Case receiving R0 gastrectomy after confirmation of P0 and CY0 following intensive chemotherapy. (PNG 40 KB) [file 12957_2013_1812_MOESM4_ESM.png]
